# Research Progress of Graphene and Its Derivatives towards Exhaled Breath Analysis

**DOI:** 10.3390/bios12020048

**Published:** 2022-01-18

**Authors:** Xinxiu Yang, Hong Chi, Yong Tian, Tianduo Li, Yaoguang Wang

**Affiliations:** Shandong Provincial Key Laboratory of Molecular Engineering, School of Chemistry and Chemical Engineering, Qilu University of Technology (Shandong Academy of Sciences), Jinan 250353, China; 17853727993@163.com (X.Y.); yongtian131@163.com (Y.T.); ylpt6296@vip.163.com (T.L.)

**Keywords:** graphene, gas sensing, biomarkers, charge transfer, colorimetry

## Abstract

The metabolic process of the human body produces a large number of gaseous biomarkers. The tracking and monitoring of certain diseases can be achieved through the detection of these markers. Due to the superior specific surface area, large functional groups, good optical transparency, conductivity and interlayer spacing, graphene, and its derivatives are widely used in gas sensing. Herein, the development of graphene and its derivatives in gas-phase biomarker detection was reviewed in terms of the detection principle and the latest detection methods and applications in several common gases, etc. Finally, we summarized the commonly used materials, preparation methods, response mechanisms for NO, NH_3_, H_2_S, and volatile organic gas VOCs, and other gas detection, and proposed the challenges and prospective applications in this field.

## 1. Introduction

The development of malignant tumors in the body changes the metabolic process, and metabolites can be excreted from the body in various ways. For example, there are volatile and semi-volatile metabolites in exhaled gas. Therefore, the analysis of some specific metabolites in the exhaled air can be used as evidence for diagnosing possible diseases in the human body [1,2,3]. The breath of a healthy person is primarily composed of nitrogen, carbon dioxide, unconsumed oxygen, and water vapor. For example, the detection of nitric oxide (NO) in the breath indicates that the patient has chronic obstructive pulmonary disease [4,5]. The production of ammonia gas (NH_3_) is usually associated with renal dysfunction [6,7]. Hydrogen sulfide (H_2_S), as a volatile sulfide compound (VSC), is a metabolite of microorganisms in the tongue coating, and is considered to be the leading cause of inflammation in the mouth [8]. There are more than 100 additional gas components with different concentrations in different patients. Table 1 summarizes the relationship between the content of biomarkers of some diseases in the exhaled breath of normal humans and patients.

Graphene (G) and its derivatives such as graphene oxide (GO) and reduced graphene oxide (rGO), etc. have been reported to show good sensing performance and are promising gas-sensitive materials. This is due to their excellent specific surface area (2630 m^2^/g), high carrier mobility (2 × 10^5^ cm^2^/Vos), and good thermal stability (oxidation resistance 650 °C) [22,23,24,25]. Many oxygen-containing functional groups (-OH, -COOH, -O, -C=O) on the surface of graphene and its derivatives endow them with vital gas adsorbing ability via potent polar effect. After adsorbing gas, graphene can adjust its own electronic distribution, change the local carrier concentration, and cause a stepped difference in resistance. The degree of resistivity differences caused by different gases is affected by the type of gas. An electron acceptor (such as NO_2_, H_2_O, I_2_) or an electron donor (such as NH_3_, CO, ethanol) will show different responsiveness characteristics, providing the scientific basis for researchers to develop graphene-based medical gas sensors [26]. To expand the optical sensing of GOs, Mei et al. developed fluorescent graphenes by covalent surface modification of graphene using alkyl amines. The surface defects induced by alkyl amines which changed the energy levels of the GO and thereby promoted blue light emissions [27]. In addition, due to the optical transparency (97.7%) and femtosecond visual response characteristics of graphene, it can adequately reflect visible light [28]. The periodic directional arrangement of the multilayer solid phase will generate visible structural color via the interference of light reflected from different interfaces. Furthermore, the ultra-thin two-dimensional (2D) single-layer structure of graphene is also beneficial to accurately studying the effect of layer spacing on the structural color.

Our research group prepared a visually colored graphene oxide (GO) film using dip-coating technology, which showed a good response to humidity in the visible light range [28]. Subsequently, the humidity sensing performance of GO film coated on a metalized polyethylene terephthalate (mPET) substrate was also discussed, providing the possibility for the application of flexible, visible devices in personal physiological state monitoring [29]. By adjusting the concentration of the GO solution, films of different thicknesses were prepared, and the colorimetric and quantitative detection of absolute ethanol gas could be realized [29]. Further study on the effect of adding polymer on selective gas detection showed that the prepared GO/polystyrene sulfonate (GO/PSS) film could achieve an ultra-fast response to NO_2_ gas at room temperature [30].

**Figure 1:**
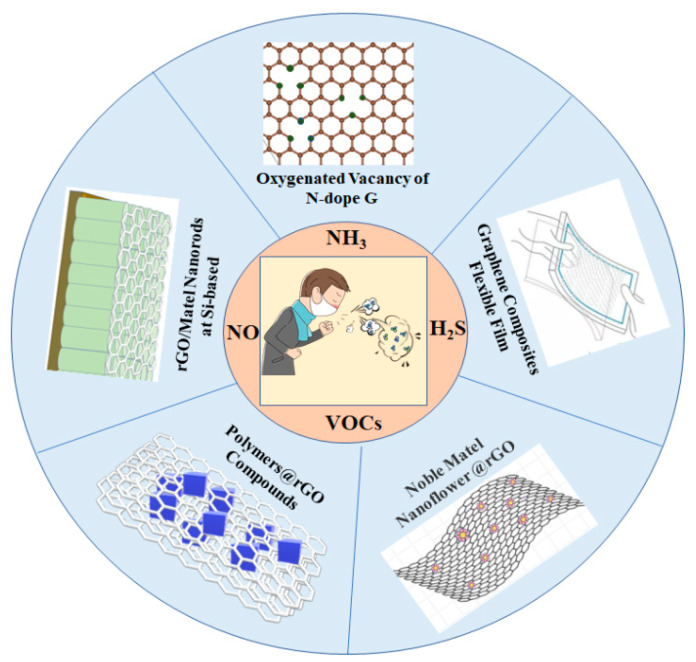
Different disease biomarkers of exhaled air identified by graphene-based composites.

In short, film interference structural color shows its superiority and importance to gas sensing due to its simple material preparation, stable physical color, and energy-saving, and in providing a new method of medical diagnosis. Herein, we focused on the recent progress of the detection and application of graphene and its derivatives in exhaled gas sensing, as shown in Figure 1, discussed from the perspectives of sensing mechanism, material preparation methods, and applications in medical health diagnosis.

## 2. Sensing Principle

### 2.1. Charge Transfer Response Mechanism

The sensing principle of graphene-based gas-sensitive materials is mainly charge transfer. That is, the sensing material itself acts as a charge acceptor or donor. When exposed to different atmospheres, charge transfer occurs between the sensitive material and the adsorbed gas. Different directions and amounts of charge transfer cause various changes in material resistance. Purging the sensitive material with inert gas can replace the adsorbed gas molecules to produce desorption. The resistance of the sensitive material can be restored to its original state.

Adsorbed oxygen means that oxygen molecules in the gas phase are adsorbed on the surface of the metal oxide due to chemical adsorption. In the operating temperature range (200–500 °C), many metal oxide materials respond to gas by adsorbing oxygen or cooperating with oxygen. In the gas-sensing mechanism, negative oxygen ions such as O_2_^−^, O^−^, and O_2_^−^ were formed on the surface of the metal oxide and worked as electron donors to provide electrons to the adsorbed gas, resulting in an increase in the conductivity of the metal oxide material and a decrease in resistance [30]. The adsorption of oxygen plays a vital role in metal oxide gas sensing materials such as ZnO, SnO_2_, etc. [31].

$$O_{2}+e^{-}\to O_{2}{}^{-}$$

$$H_{2}S+\frac{3}{2}O_{2}{}^{-}\to H_{2}S+SO_{2}+\frac{3}{2}e^{-}\;$$

**Figure 2:**
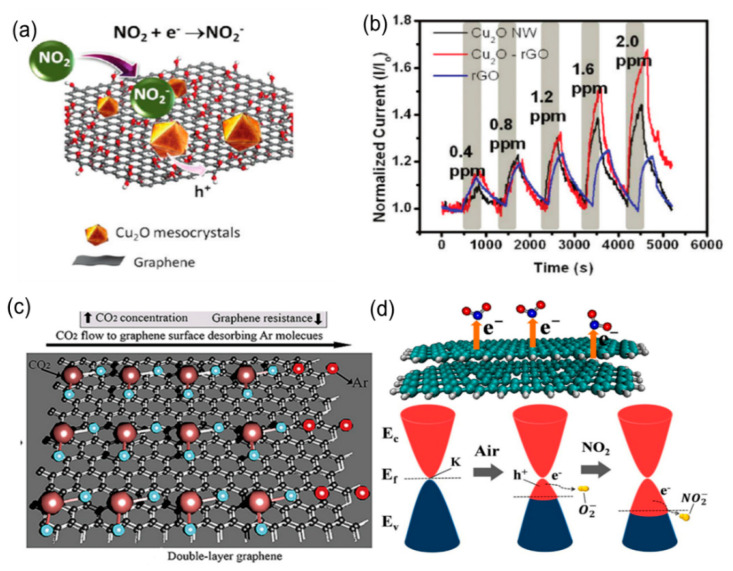
(**a**) Schematic diagram of Cu_2_O–rGO sensing mechanism for NO_2_. (**b**) Relative current changes of Cu_2_O NW, rGO–Cu_2_O, and rGO based devices when exposing to different concentrations of NO_2_ [32]. Copyright (2021), used with permission from the American Chemical Society. (**c**) Schematic diagram of carbon dioxide adsorption/desorption with argon purging the graphene surface [33]. Copyright (2018), used with permission from Elsevier. (**d**) Schematic diagram of the sensing mechanism and energy band change of the charge transfer between the double-layer graphene gas-sensitive material and NO_2_ [34]. Copyright (2017), used with permission from Elsevier.

For example, Deng et al. prepared Cu_2_O nanowire–rGO (Cu_2_O NW–rGO) composites by using rGO to induce and stabilize the superstructure [32]. As shown in Figure 2a, when the material is contacted with oxidizing NO_2_ gas, the gas molecules will receive electrons from the “activated” surface oxygen ions, promoting an increase in hole conductivity in the Cu_2_O device.

$$NO_{2}+e^{-}\to NO_{2}{}^{-}$$

When detecting NO_2_, the composite material current can change significantly as compared with rGO and Cu_2_O NW (Figure 2b).

Fan et al. studied the charge transfer mechanism between CO_2_ and double-layer graphene [33]. Here, CO_2_ acts as an electron acceptor to bind onto graphene. This work qualitatively studied the influence of two different adsorbent types on the electronic structure of double-layer graphene (indicated by the difference in charge density). The adsorption of electron acceptor CO_2_ will increase the charge on the surface of the double-layer graphene and increase the conductivity. When purging with dry argon (Figure 2c), the argon molecules replace the electron acceptor CO_2_ gas molecules that adsorbed on the graphene surface, resulting in the desorption of CO_2_ molecules, thereby reducing the charge concentration on the graphene surface and causing a resistance increase. Seekaewa et al. studied the sensing mechanism of single-layer, double-layer, and multilayer graphene based on the charge transfer for NO_2_ [34]. As shown in Figure 2d, the double-layer graphene exhibits a significant Fermi-level shift and conduction bands intersect at the K point before gas adsorption. After adsorption, NO_2_ molecules that adsorbed on the graphene extract more electrons from the valence band to convert themselves into NO_2_^−^, resulting an increase in the hole density of the graphene film and a decreased Fermi level. In addition, as the number of graphene layers increases, the number of holes will also increase. Therefore, when the gas is adsorbed, the wider valence band of the double-layer and multilayer graphene will generate more electrons, resulting in a smaller resistivity. This explains why the resistance of the adsorbed *n*-type gas decreases drastically as the number of graphene layers increases.

### 2.2. Response Mechanism of Field-Effect Transistor

When the graphene sensing material is prepared in a field-effect transistor (FET), the sensing principle is mainly dependent on the resistivity (R) and the back gate voltage (V_bg_), and the peak value corresponds to the neutral point of the charge (CNP peak), which is defined by the Fermi level across the Dirac point, and the total charge in graphene at the Dirac point should be zero. Ideally, the movement of the CNP peak in the sensor is caused by the doping of adsorbed molecules. Under the effects of air and water, the actual graphene FET sensor usually exhibits lags in its sensing performance [35,36].

**Figure 3:**
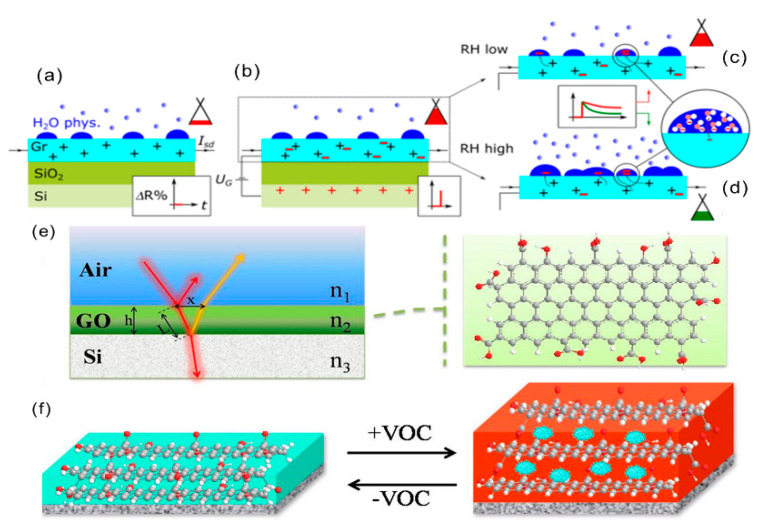
(**a**) Schematic diagram of the initial state of a graphene FET after physical interaction of electrons with water; (**b**) electrons change immediately after the gate voltage is applied; (**c**,**d**) schematic diagram of the state when a more extended grid voltage is applied (**c**) at lower and (**d**) at higher relative humidity [37]. Copyright (2020), used with permission from Elsevier. (**e**) The principle of thin-film interference structure; (**f**) schematic diagram of VOC gas–sensing mechanism based on gas intercalation and visual response [26]. Copyright (2018), used with permission from Elsevier.

Bartošík et al. found that the p-doping of graphene FET sensors is caused by the capture of graphene due to the chemical adsorption of water on the graphene–SiO_2_ interface [37]. As shown in Figure 3a, when water molecules are physically adsorbed on the graphene surface at higher relative humidity (RH%), the water molecules are captured by graphene due to chemical adsorption on the graphene–SiO_2_ interface. The Fermi level in the graphene cone is lower than the Dirac point (Figure 3b). At this time, the primary carrier of the source-drain current (I_sd_) is the hole. After the positive gate voltage is applied, the electrons immediately flow into the graphene, inducing the Fermi level to move upward to a position close to the Dirac point, resulting in a significant increase in resistance. When the electrons in the graphene are gradually captured by physically adsorbed water, they diffuse more profoundly into the water droplets (Figure 3c). In the continuous process (Figure 3d), the Fermi level moves further down from the Dirac point, leading to a decrease in resistance. Because the capture and diffusion of electrons in water is slow, therefore, under higher RH%, the greater the amount of physically adsorbed water, the more electrons can be captured, and the faster the resistance drops.

### 2.3. Thin-Film Interference Response Mechanism

The physical color of the thin-film interference structure is produced due to the regular arrangement of photonic crystals (PC) [38]. Dielectric materials with different refractive indexes will selectively reflect light of specific wavelengths due to the different periodic arrangements of the photonic crystals. The photonic crystal includes dielectric microstructures with one-dimensional, two-dimensional, or three-dimensional periodicity [34,39]. Sensors based on photonic crystals (PC) do not require any additional electrical test systems. They exhibit vivid color conversion and possess high sensitivity and good reversibility, making them a promising real-time gas monitoring sensor material [40,41]. Gas sensor materials based on thin-film interference include polymer electrolytes [42,43], cholesteric liquid crystal [44], graphene oxide, and a composite material containing cobalt chloride [45]. Our group reported the preparation of a GO-based thin-film interference gas sensor with optical colorimetric sensing characteristics. A simple dip-coating technique was used to prepare GO film with visual colorimetric properties [26]. By adjusting the solution concentration, the above film could show other colors. As shown in Figure 3e, the light reflected at different interfaces overlaps the light path and phase interference, showing visual color. GO is rich in negatively charged carboxyl groups. The hydroxyl groups in ethanol with a lone pair of electrons can be easily diffused and inserted into the GO layers. As shown in Figure 3f, it can be understood that the intercalation leads to an increase in the interlayer spacing and thus produces different visualized colors.

## 3. Gas Monitoring for Medical Diagnosis

### 3.1. Nitric Oxide

Nitric oxide (NO) is synthesized by L-arginine through nitric oxide synthase (NOS) in various cells such as neutrophils, red blood cells, endothelial cells, macrophages, and nerve cells. The determination of NO content in exhaled air has been used as a marker for judging the degree of acute respiratory infection and inflammation in respiratory diseases [12]. The presence of NO in exhaled gases can be used for rapid detection of lung infections [46], including new coronary pneumonia [47], asthma [48], allergic rhinitis [49] severe acute respiratory syndrome (SARS) [50], and chronic obstructive pulmonary disease [51]. At the same time, continuous monitoring of NO is also essential for coronary heart disease [52], Alzheimer’s disease [53], and mental illness [54]. Jiang et al. reported on a chlorinated graphene FET and its application in monitoring of NO under specified physiological requirements [55]. To facilitate naked eye-based on-site detection, Zhou et al. recently developed ratiometric fluorescent probes with distinctive color changes to monitor exhaled nitric oxide to indicate the clinical course of asthma [56]. The fluorescent nanoprobe was also integrated with smartphone analysis for rapid and sensitive detection of exhaled NO.

**Figure 4:**
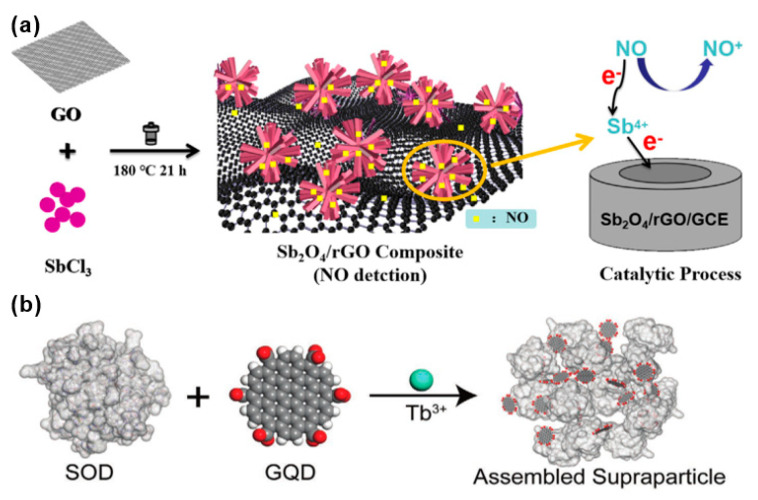
(**a**) Synthetic route of antimony tetroxide (Sb_2_O_4_) nanoflower/rGO nanocomposite [57]. Copyright (2021), used with permission from Elsevier. (**b**) Schematic diagram of the process of monodisperse superparticles (SPs) prepared by coordinating bridged graphene quantum dots (GQDs) and superoxide dismutase (SOD) using terbium ion as metal [59]. Copyright (2021), used with permission from Wiley.

Deng et al. synthesized an antimony tetroxide (Sb_2_O_4_) nanoflower/rGO nanocomposite material using a simple and environmentally friendly solvothermal method [57]. As shown in Figure 4a, the combination of highly electroactive Sb_2_O_4_ nanoflowers and rGO with a larger specific surface area shows a synergistic effect on the recognition of NO. It can detect the NO released by living cells in real time, effectively distinguishing normal and tumor cells. In this case, the reactive Sb^4+^ and rGO help to achieve rapid electron transfer and the nanoflower structure provides a three-dimensional high-speed channel for NO, making the distance across which the NO must travel to reach the reaction center very short, and thereby obtaining a high mass transfer rate. The Sb_2_O_4_/rGO hybridization has a low detection limit for NO in the range of 3.98 nm~0.772 μM (3.98 nM). Real-time detection of NO found that the NO released by tumor cells is five times that of normal skin cells. Mathew et al. prepared a Pt-electrochemical reduction graphene oxide (erGO) modified glassy carbon electrode (GCE) by using a simple green synthesis method using a sequential electrochemical process [58]. The effect of the preparation route on the NO sensor was also studied. Pt and erGO were deposited on the GCE in different orders. The material was prepared by coating Pt NPs on the erGO surface as SQ-I was Pt–erGO, and by coating erGO on the Pt NPs surface as SQ-II was erGO–Pt. Different preparation routes have significant differences in sensitivity, accuracy, selectivity, and stability. Compared with bare GCE and SQ-II electrodes, the SQ-I electrode showed a lower charge transfer resistance, and the electron transfer rate on the surface of the SQ-I electrode was faster than that of the SQ-II electrode. Pt nanoparticles on the surface of a Pt-modified erGO matrix electrode could coordinate with NO and effectively transfer electrons, thus improving the sensitivity to NO. Thus, the SQ-I electrode is more suitable for electrochemical detection of NO. Compared with a bare electrode, the current response of Pt–erGO (SQ-I) to NO is increased by ~15 times. The sensitivity of Pt–erGO/GCE to NO is 8.40 μA^−1^ cm^−2^μM^−1^; the linear range is 0.25–40 μM, the lowest limit is 52 nM, and the response time is 0.7 s. In addition, it has good selectivity for NO. Pt–erGO/GCE is expected to be a candidate material for future clinical application due to its good stability, repeatability, and practicability.

Inspired by self-limiting nano-components, Qu et al. assembled graphene quantum dots (GQDs) and superoxide dismutase (SOD) into monodisperse superparticles (SPs) [59]. As shown in Figure 4b, graphene quantum dots (GQDs) and superoxide dismutase (SOD) were assembled to synthesize GQD–Tb^3+^–SOD SPs, and were coated on a glass plate for NO sensing test. Superoxide dismutase (SOD) can alternately catalyze the dismutation of superoxide anion free radicals (O^2−^), usually with copper as the active center. When Cu(II) in SOD is reduced to Cu(I), the fluorescence of the optically active component Tb^3+^ fluorophore is enhanced, which is tightly sandwiched by protein and GQD. Tb^3+^ combines graphene quantum dots (GQDs) and SOD to realize effective energy transfer, thereby “turning on” detection of NO, and realizing reliable monitoring as low as 600 molecules/mL. It is verified that the sensor can achieve ultra-sensitive detection of NO with a concentration as low as 10 × 10^–12^ M. The sensor opens up a new direction in using self-limiting nano-components. The high sensitivity and non-invasiveness of SP detection means it is widely used in home health monitoring.

**Figure 5:**
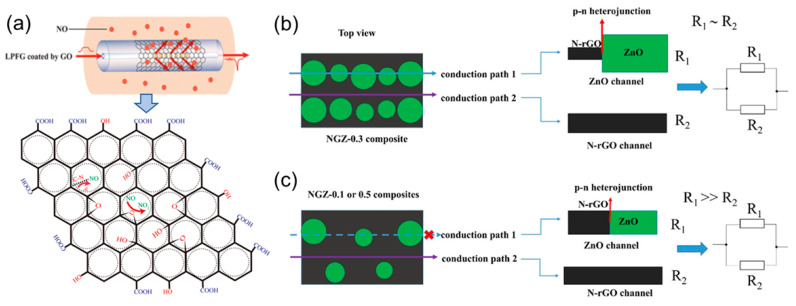
(**a**) The structure of GO–LPFG and the response mechanism to NO [60]. Copyright (2019), used with permission from Elsevier. (**b**) Physical model of NGZ–0.3 (the volume of NH_4_OH is 0.3 mL) composite material N–rGO/ZnO (NGZ); (**c**) physical model of NGZ–0.1 or NGZ–0.5 (NH4OH volume 0.1 and 0.5 mL) composite material [61]. Copyright (2020), used with permission from Elsevier.

Xu et al. constructed an ultra-sensitive NO gas sensor by long-period fiber grating (LFPG) which was covered with GO on a long-period fiber grating (LFPG) that had good sensitivity by refractive index (SRI) changes [60]. The GO was coated on the long-period fiber grating (LPFG) (Figure 5a). It can be seen that NO adsorption on the GO depends on two adsorption processes. NO is oxidized by epoxy groups to generate nitrogen dioxide. These complex processes cause NO to be completely removed from the GO surface. The sensor can identify 0–400 ppm NO with high sensitivity. To create more active centers, adjust the band gap of the composite material, and effectively improve the catalytic activity, Qiu et al. used metal oxide nanocrystals (ZnO) to dope redox graphene with nitrogen (N–rGO). These promote the interaction with the attached metal oxides, create more active centers for gas adsorption, and reduce the hydrolysis rate of Zn(OAc)_2_ by adding different amounts of NH_4_OH dropwise to achieve controllable nucleation. In the experiment, the volume of NH_4_OH was controlled to be 0.1, 0.3, and 0.5 mL, and the corresponding products were named NGZ–0.1, NGZ–0.3, and NGZ–0.5 to obtain N–rGO/ZnO (NGZ) hybrid material [61]. At low temperatures, the gas-sensitive response of ZnO nanocrystals on the surface of N–rGO to NO is much better than that of ZnO microcrystals aggregated or formed on the surface of N–rGO alone. The resistance of the zinc oxide channel with a heterojunction in Conduction path 1 in Figure 5b,c is much larger than that of the N–RGO channel in Conduction path 2 (R1 ≫ R2). Because metal oxide nanoparticles with a smaller particle size have a p–n heterojunction in NGZ–0.3, which has a strong adsorption effect on NO, the sensitivity of NGZ–0.3 to NO is much greater than that of NGZ–0.1 and NGZ–0.5. Table 2 summarizes the progress of NO-sensing materials, detection limits, and response/recovery time in recent years.

### 3.2. Nitrogen Hydride

The production of NH_3_ in the human body is related to protein metabolism. Proteins are degraded into non-storable amino acids to be used or metabolized, which leads to the formation of NH_3_ [38,56]. Ammonia (NH_3_) itself is a toxic and irritating gas. Its accumulation in the human body promotes acidosis in the blood, causes enzyme denaturation, and ultimately leads to death. When the concentration of exhaled ammonia exceeds 2935 ppb, it means that the kidney urea cycle is out of balance. Due to the gas exchange between blood, alveoli, and air, a small amount of ammonia is exhaled in the breath. The main mechanism of ammonia metabolism is the excretion of the urine after the detoxification process, which is the renal urea cycle. In this process, NH_3_ is converted into the less toxic soluble compound urea [13]. If the kidneys cannot filter the urea in the blood, urea will enter the body’s gas circulation with the blood, and the NH_3_ concentration in the breath will increase [13,69,70]. This is why patients with chronic kidney disease (CKD) exhale ammonia gas.

Compared with G, oxygen-containing groups on the surface of GO can enhance the gas sensitivity by interacting with polar gas molecules. There are still some oxygen-containing functional groups on the surface of rGO. To distinguish the influence of polar water molecules on NH_3_ detection, Kim et al. studied the response of graphene gas sensors under two different humidity (RH%) conditions [63]. A passivation layer of polystyrene (PS) brush was used on the surface of silicon dioxide to cover the hydroxyl groups in SiO_2_/Si.

When the humidity increased from 5% to 50%, the response of graphene field-effect transistors (FETs) on the bare brush and PS brush to NH_3_ decreased slightly (by about 9%). The weakening of the response under relative humidity indicates that the presence of water molecules partially limits the adsorption of NH_3_. The long-term sensing behavior of the graphene sensor under NH_3_, CO_2_, and relative humidity conditions was monitored through saturation experiments, and the cross-sensitivity of the graphene sensor under CO_2_ and comparable humidity conditions was verified. The key reason for the excellent cross-sensitivity of the graphene FET gas sensor on the PS brush is the reduction in substrate-induced doping.

**Figure 6:**
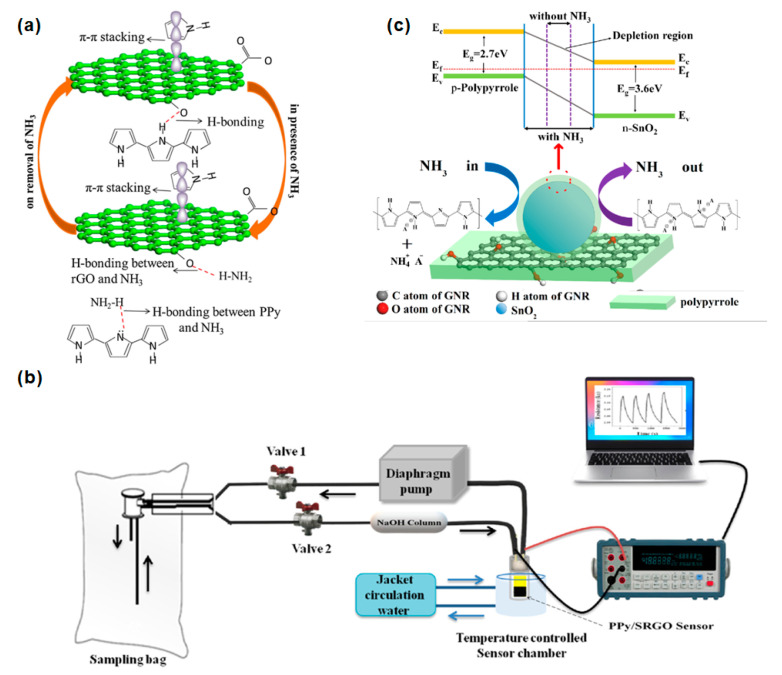
(**a**) Schematic diagram of the interaction between NH_3_ and PPy/rGO [64]. Copyright (2017), used with permission from Elsevier. (**b**) Device of PPy/srGO nanocomposite gas sensor [7]. Copyright (2021), used with permission from Elsevier. (**c**) The energy band diagram and sensing mechanism of the PPy/SnO_2_/GNR nanocomposite sensor [65]. Copyright (2021), used with permission from Elsevier.

Sun et al. used a more straightforward method to prepare a polypyrrole (PPy) and rGO composite material through covalent bonding via in-situ chemical oxidation polymerization [64]. The PPy/rGO was coated on a flexible PET film to construct a flexible and cost-effective NH_3_ sensing film. The 5 wt. % rGO–PPy hybrid sensor not only had the highest response sensitivity to 1–10 ppm NH_3_, but the response sensitivity was 2.5 times that of pure PPy. Figure 6a shows that the improvement in gas sensitivity may be attributed to the π–π stacking and the formation of hydrogen bonds between PPy and rGO, resulting in a larger surface area and fast carrier transport after the two are recombined. Shahmoradi et al. used an in-situ chemical oxidative polymerization method to prepare polypyrrole (PPy) composites with graphene oxide (GO), reduced graphene oxide (rGO), or p-benzenesulfonic acid sulfonated reduced graphene oxide (srGO) [7]. The sensor response process is shown in Figure 6b. The effect of the working temperature of different nanocomposite materials at four temperatures (28, 40, 50, and 60 °C) on the sensor’s performance was studied. The PPy/srGO sensor showed the highest detection sensitivity, which was 0.20 ppb~12 ppm NH_3_. The response principle is attributed to the charge transfer mechanism between NH_3_ and the nanocomposite surface. The electron transfer process of NH_3_ molecules to the surface of the p-type polypyrrole sensor is depleted, resulting in an increase in resistance. On the other hand, p-type rGO is donated to electrons. When the PPy/srGO sensor is exposed to NH_3_ gas, a hydrogen bond interaction was generated between NH_3_ and bare PPy and also between NH_3_ and srGO; thereby the electrical resistance was increased. Hsieh et al. further improved the hybrid system of PPy and graphene. They prepared a polypyrrole (PPy)/tin oxide (SnO_2_)/graphene nanoribbon (GNR) ternary nanocomposite by using an in-situ chemical oxidation polymerization method [65]. The results showed that the response sensitivity of the PPy/SnO_2_/GNR sensor containing 3 wt% SnO_2_ nanoparticles in 1 ppm NH_3_ is about three times that of pure PPy. Figure 6c shows that the PPy-coated SnO_2_ and GNR can form covalent bonds. The larger specific surface area contributed by SnO_2_ and GNR could further enhance the contact site with PPy and provide many adsorption sites for NH_3_ gas. The band gaps of PPy and SnO_2_ are 2.81 eV and 3.709 eV, respectively. Thus, the p–n heterojunction formed between p-type PPy and n-type SnO_2_ benefits the formation of a self-established depletion layer electric field at the PPy/SnO_2_ heterojunction.

**Figure 7:**
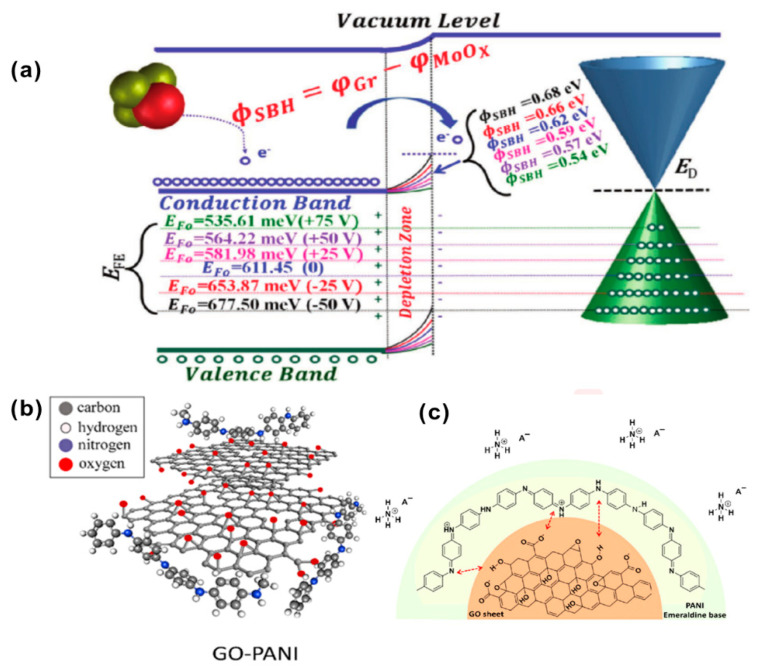
(**a**) Fermi-level tuning diagram in MoOx/graphene composite field-effect transistor sensor (D100) (back gate voltage V_GS_ from −50 to +75 V, electron transfer from NH3 to p-type graphene) [66]. Copyright (2020), used with permission from Elsevier. (**b**) The expanded coil when PANI nanocomposites were pinned to the GO sheets; (**c**) the adsorption mechanism of GO–PANI in ammonia [68]. Copyright (2021), used with permission from Elsevier.

Falak et al. used a simple and contamination-free shadow mask approach to hybridize an ultra-thin MoO_x_ layer with a single layer of graphene with different molybdenum oxide coverage [66]. The MoO_x_/GFET (graphene field-effect tube) showed a response recovery time of 356 s to 12 ppm NH_3_, and a lower detection limit of 310 ppb. The excellent sensing and recovery performance of the MoO_x_/GFET sensor is mainly attributed to the effective adjustment of the height of the Schottky barrier. The energy band in Figure 7a indicates the changes in hole density and Fermi energy caused by NH_3_ exposure. Under the bias voltage from −50 V to +75 V, the positive change in the back-gate voltage (V_GS_) causes the Fermi energy valence band to move upward to make it closer to the energy level of NH_3_, resulting in a smaller energy difference and less charge transfer between NH_3_ and p-type GFET sensors.

Javadian-Sara et al. explored a microwave-based open-loop resonator (SRR) sensor based on the nanocomposite prepared by the in-situ polymerization of polyaniline (PANI) on the surface of GO [68], as shown in Figure 7b. At room temperature, it has a high sensitivity of 0.038 dB ppm^−1^ to low concentration (1–25 ppm) ammonia, a response/recovery time of 150/400 s, and a sensitivity of 0.0045 dB ppm^−1^ to high concentrations (>25 ppm). As shown in Figure 7c, when the GO(10%)–PANI nanocomposite is exposed to ammonia gas, due to the acid-base interaction between PANI and ammonia gas, the PANI changes from a conductive form to a non-conductive state. Ammonia gas acts as a base to reduce the aniline ion R–NH^+^ carriers of PANI through deprotonation, showing a strong de-doping effect and enhancing the internal resistance of PANI. The prepared sensor can selectively detect ammonia in the atmosphere of other higher concentrations of dangerous gases and across a wider range of RH% (15–90%). The response signal can be repeated after 30 days with less than 0.32% variation.

The high efficiency of graphene-based sensor components can be achieved by designing and optimizing their preparation processes, but graphene-based sensors for the physiological analysis of ammonia in breath are still being studied.

### 3.3. Sulfide Gas

The cause of bad breath is multifactorial, involving various body organs (mouth, lungs, and stomach). About 90% of all types of bad breath can be classified as intraoral bad breath, which stems from oral-related pathological conditions (dental periodontitis and gingivitis) and physiological characteristics, especially the microbial coating on the tongue [71,72]. The microorganisms in the tongue coating produce a variety of metabolites, including volatile sulfides (VSCs), such as methyl mercaptan (CH_3_SH), hydrogen sulfide (H_2_S), and dimethyl sulfide (CH_3_SCH_3_) [8,73,74].

**Figure 8:**
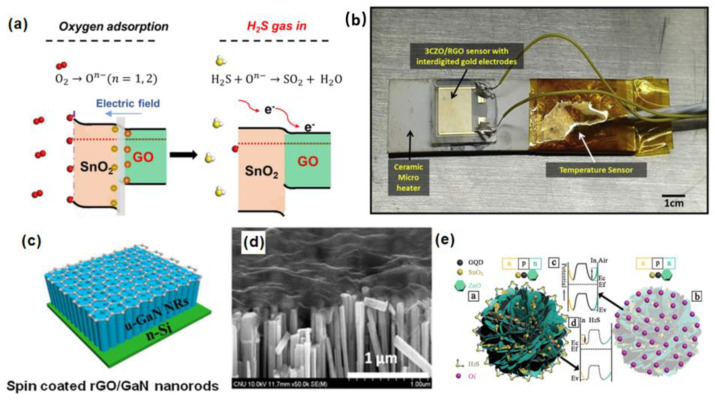
(**a**) Schematic diagram of H_2_S adsorption mechanism based on SnO_2_@GO gas sensor [75]. Copyright (2020), used with permission from Elsevier. (**b**) CZO/rGO sensor structure [76]. Copyright (2020), used with permission from Elsevier. (**c**) Schematic diagram of rGO)/GaN nanorods (NRS); (**d**) scanning electron microscope (SEM) image of rGO/GaN nanorod (NRS) composite material [77]. Copyright (2020), used with permission from Wiley. (**e**) Schematic diagram of band configuration at the interface of the GQD–SnO_2_/ZnO [81]. Copyright (2021), used with permission from the American Chemical Society.

Song et al. loaded SnO_2_ quantum wires (QWS) with a diameter of less than 4 nm onto graphene oxide (GO) through simple mechanical mixing [75]. The prepared sensor has a fast dynamic response to the detection of ppb H_2_S at 70 °C and 85% RH%. The lower limit can reach 61 ppb with good response/recovery performance. In addition, the GO–loaded SnO_2_ QWS has solution processability after oleic acid (OA) and oleylamine (OLM) treatment, and it can be loaded on a paper base flexible H_2_S sensor. As shown in Figure 8a, the adsorption of oxygen causes electrons in the conduction band of the SnO_2_ QW to be deprived, causing the energy band to bend and increasing the resistance. The H_2_S gas can react with the oxygen adsorbed on the surface, and it is oxidized to sulfur dioxide, making the electrons return to the SnO_2_ QW. In this case, it helps to adjust the surface state of the band structure, so that SnO_2_ QWs generate more oxygen adsorption sites. At the same time, GO acts as an electron migration channel, which improves the sensitivity of selection. Shewale et al. used a simple air-jet deposition method to uniformly modify GO on copper-doped zinc oxide (CZO) nanostructured films [76]. As shown in Figure 8b, the 3CZO/rGO sensor doped with ZnO/rGO (mass fraction of 3 wt% copper nitrate aqueous solution) is fixed on the ceramic microheater with thermal paste. To accurately measure the temperature of the gas sensor, a highly sensitive temperature sensor is installed on the surface of the microheater. The synthesized 3CZO/rGO nanocomposite sensor shows better gas sensitivity to H_2_S at room temperature (24 °C) than undoped ZnO/rGO, and a better response to H_2_S. The response and recovery time to 100 ppm H_2_S are, respectively, 14 s and 32 s, which dramatically improves the potential application of nanocomposite sensors in medical diagnosis. The recent applications of other graphene and its derivatives in the detection of H2S gas biomarkers were summarized in Table 3.

**Table 3:** The detection range and response/recovery time of graphene composites to H_2_S gas.

Material	Gas	Detection Limit	Response/Recover Time (s)	References
SnO_2_@GO	H_2_S	200 ppb	9/23 (54%RH)	[75]
	H_2_S		6/21 (93.6%RH)	
ZnO/rGO	H_2_S	136 ppb	14/32	[76]
rGO/GaN	H_2_S	100 ppm	~800	[77]
(rGO) –NiO(NiOBNG)	H_2_S	1 ppm	31/49	[78]
	H_2_S	20 ppm	38/44	
	H_2_S	50 ppm	28/75	
β–Ga_2_O_3_/rGO	H_2_S	3 ppm	N.A.	[79]
WO_3_/rGO	H_2_S	32.7 ppb	340/180	[80]
GQD–SnO_2_ QNP/ZnO	H_2_S	0.1 ppm	14/13	[81]

N.A.: not available.

Reddeppa et al. reported a rGO/GaN nanorod (NRs) hybrid system for hydrogen (H_2_) and hydrogen sulfide (H_2_S) gas sensing at room temperature [77]. Figure 8c shows the prepared (rGO)/GaN NRs hybrids. As shown in Figure 8d, the surface of the GaN nanorods has a wavy structure without the rGO film, and the spin-coated reduced graphene oxide layer does not penetrate between the nanorods. Compared with pure GaN NRs, rGO/GaN NRs have better gas sensitivity.

Shanmugasundaram et al. prepared a hierarchical mesoporous nickel oxide (NiO) nanodisk and boron nitrogen co-doped rGO–NiO (NiOBNG) nanodisk composite [78]. The NiOBNG sensor has a sensor response of 82% to 100 ppm H_2_S, and the detection limit is 24 ppb. At 150 °C, the NiOBNG showed good selectivity to 20 ppm hydrogen sulfide compared with 100 ppm NH_3_, H_2_, CO, nitrous oxide, ethanol, and methanol. The responsiveness of the NiOBNG sensor has been increased by two times and three times, respectively, which is attributed to the catalytic active centers of boron and nitrogen-doped rGO. Deji et al. simulated the adsorption of hydrogen sulfide (H_2_S) gas on the surface of undoped armchair graphene nanoribbon (AGNRs) and doped osmium graphene nanoribbon (Os–AGNRs) [81]. Using DFT theory, the adsorption energy of osmium-doped AGNRs, unilateral doping, center doping, and bilateral doping were studied. The adsorption energy results show that the adsorption energy of undoped AGNRs is about −0.25 eV and it is not sensitive to H_2_S, whereas the adsorption energies of unilateral and bilateral doped AGNRs are −6.67 eV and −9.67 eV, respectively. The density of states analysis shows that the electronic properties of the bilateral doped Os–AGNRs have changed significantly. In addition, the material changes from the original semiconductor to metal after adsorbing H_2_S molecules in the case of doping on both sides. This drastically changing electronic behavior gives the new double-edge doped Os–AGNRs potential for H_2_S gas adsorption and sensing applications. Shao et al. reported a self-assembled graphene quantum dot (GQD) functional hierarchical SnO_2_ nanoparticle (SnO_2_ QNP)/zinc oxide nanosheet (ZnO) [82]. The strong synergistic effect between p-type GQD and n-type SnO_2_ and ZnO makes the p–n heterojunction effectively increase the resistance caused by the change of oxygen adsorption. Compared with the original ZnO and SnO_2_/ZnO sensors, the SnO_2_ QNP/ZnO nanostructures modified by GQD have a very high response to H_2_S: the response S to 0.1 ppm H_2_S is 15.9 (the resistance of the sensor that is exposed to air (R_a_) divided by the resistance when it is exposed to the target analyte (R_g_)). They also have a fast response/recovery time (14/13 s). 

### 3.4. VOCs

Volatile organic compounds (VOCs) can be generated through various biochemical pathways. The detection of VOC biomarkers can be realized by measuring changes in the electrical, optical, chemical, and chromatic properties of sensitive materials that interact with VOC molecules [83]. Probiotics can regulate intestinal problems and maintain the body’s intestinal homeostasis. It is possible to diagnose the balance of human probiotics by detecting changes in the concentration of trimethylamine (TMA) in exhalation [9]. Yu et al. reported the real-time monitoring of TMA in respiration based on the 11–mercaptoundecanoic acid (11–MUA)—Au nanoparticles (AuNPs)/rGO composite material microgravimetric method [84]. The sensor shows that the lower limit of detection is 5 ppm, and the response time is 30 s. Hexanal and 5-methylundecane are biomarkers of multiple sclerosis (such as Alzheimer’s disease (AD) and Parkinson’s disease (PD)) [85]. Mazzatenta et al. reported the real-time detection of AD fingerprints in exhalation. A significant change in the accumulated VOC fingerprints was detected in the breath of an AD patient [86]. It was proposed that the expiratory VOC was based on the biomarker concentration that gradually changes with the progression of the disease to distinguish the substages of AD. A sensor array based on single-walled carbon nanotubes and polycyclic aromatic hydrocarbons was used to detect VOC biomarkers of AD. Direct analysis of breath showed 85.3% sensitivity, 70.6% specificity, and 80.4% accuracy. The recent applications of other graphene and its derivatives in the detection of organic volatile gases that can be used as markers for cancer and other diseases were summarized in Table 4.

**Figure 9:**
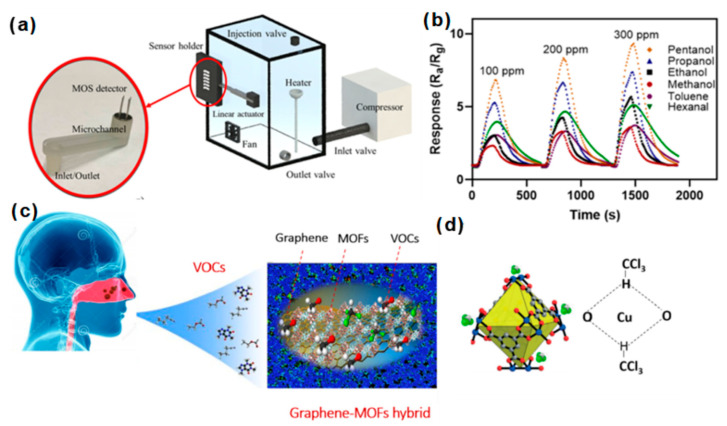
(**a**) Schematic illustration of the microfluidic gas sensor; (**b**) the sensing intensity of the microfluidic gas sensor for different VOCs [87]. Copyright (2022), used with permission from the American Chemical Society. (**c**) Response of graphene and MOF hybrid nanocomposite on exhaled gas; (**d**) the schematic diagram of the hydrogen bond affinity between copper benzene–1,3,5–tricarboxylate (Cu–BTC) and chloroform molecules [93]. Copyright (2020), used with permission from the American Chemical Society.

Ghazi et al. developed a GO microfluidic gas sensor by: (1) 3D printing microchannels coated with a 5-µm-thick parylene carbon layer (propylene polymer). Then, the xylene was used to provide a chemically inert barrier layer. (2) The modified microchannel was coated with GO to improve the specific surface area to volume ratio of the micro-features [87]. The performance was improved. The sensing setup of the microfluidic gas sensor is shown in Figure 9a. Compared with ordinary gas sensors, its selectivity to toluene, ethanol, methanol, pentanol, propanol, and hexane increased by 64.4% on average (Figure 9b).

It is understood that the abnormal concentrations of toluene exhaled by lung cancer patients are in the hundreds of ppb, whereas the normal concentration is about 1–18 ppb. Pargoletti et al. synthesized a SnxTi_1−x_O_2_/GO–based material through a simple hydrothermal method, where x is the content ratio of tin [90]. When using 32:1 SnO_2_/GO and 32:1 TiO_2_/GO, there is excellent selectivity to acetone at a level of 100 ppb. However, when compared with titanium, when a solid solution with a higher tin content (such as 32:1 Sn_0.55_Ti_0.45_O_2_/GO) is used, it shows a higher performance for the larger non-polar molecule toluene at 350 °C. Tung et al. prepared three different graphene-MOF hybrid nanocomposites, including

Coppere–benzene–1,3,5–tricarboxylate (PG–Cu BTC)), zirconium 1,4–dicarboxybenzene 1,4–dicarboxylate (PG–UIO 66) and 2-methylimidazole zinc salt (PG–ZIF 8). They used this for chemoresistance sensing of VOC biomarkers [93]. As shown in Figure 9c, the PG–Cu–BTC sensor was tested against VOC biomarkers, and it showed good selectivity to chloroform. The selectivity principle (Figure 9d) is attributed to the hydrogen bond between the chloroform molecule and Cu-BTC.

Diabetes mellitus (DM) is a metabolic disease characterized by high blood sugar caused by insufficient insulin secretion (type I DM), insufficient insulin action (type II DM), or both. Due to hyperglycemia conditions (high blood sugar levels when fasting GI is greater than 7 mmol/L), the excess sugar cannot be metabolized, which induces the decarboxylation of acetoacetate and the dehydrogenation of isopropanol. Large amounts of isopropanol in the liver can produce acetone [94]. The acetone produced in the blood can diffuse through the lungs to the airways, because acetone has a blood/air partition coefficient of 341 at body temperature, and diffuses through urine. Therefore, diabetes can be diagnosed by testing the concentration of acetone in the breath at present [95]. Studies have determined that the average concentration of acetone in the breath of healthy people is 300–900 ppb, and the abnormal concentration of acetone in the breath of diabetes patients exceeds 1800 ppb.

Kalidoss et al. discussed the influence of the synthesis route on material properties. The solvothermal method (GS-I) was used to prepare rGO/SnO_2_ composite material through chemical synthesis and the hydrothermal method (GS-II) was used to prepare it through mechanical mixing [89]. At a working temperature of 200 °C, GS-I has a larger specific surface area and surface energy than GS-II. Research results show that GS-I is more suitable for acetone sensing in exhalation and can clearly distinguish between health and diabetes. The response mechanism to acetone can be attributed to the synergistic effect of the rGO/SnO_2_ interface. Yempally et al. prepared a quantum resistance vapor sensor made of graphene and cellulose acetate matrix, ultrasonically mixed and reacted for 30 min [96]. The nanostructures were formed through layer-by-layer scattering to distinguish only 1 ppm of acetone vapor, which simplifies the propagation of the analyte to the nano-junction, thereby improving the sensitivity. The sensor showed good response to acetone, ethanol, methanol, and water with concentrations ranging from 1 to 100 ppm. The signal-to-noise ratio of 1 ppm of acetone is about 15 ppm, which can be used to non-invasively monitor the blood glucose level of diabetic patients. Graphene-based materials provide higher sensitivity and faster recovery in VOC sensing. Chemically modified graphene has the advantages of mass production, high yield, and easy control of function, which can be widely used in the preparation of graphene-based volatile organic compound sensors. 

## 4. Conclusions and Perspectives

Graphene-based composite smart sensors have high activity against several disease-marking gases, and have been widely studied in medical diagnosis. In this article, we reviewed composite materials based on graphene and graphene derivatives (including graphene oxide (GO), reduced graphene oxide (rGO), and sulfonated reduced graphene oxide (srGO)). Preparation methods (mechanical mixing, hydrothermal synthesis, solvothermal, vacuum annealing, post-synthesis humidity treatment technology, vapor deposition CVD, self-assembly, etc.) and the sensing performance for NO, NH_3_, H_2_S, and volatile organic gas VOCs (toluene, acetone, cyclohexane, chloroform, etc.) were also included. Gas-sensing mechanisms such as formaldehyde, ethanol, methanol, trimethylamine, etc., including the direct electron transfer mechanism, Fermi level mechanism, and color mechanism of thin-film interference mechanisms, and their applications in detecting disease-related gases were elaborated and discussed in detail. Although graphene-based sensors have high sensitivity, they still face many challenges, such as repeatability, selectivity, dispersion, and functional group stability, that need to be further optimized.

In addition, there are factors that are difficult to control in the preparation of materials: for example, it is difficult to control the number of layers, functionality, and specific chemical structure of graphene. A series of complicated processes are still required in the device manufacturing and sensing process. For example, for graphene synthesized by chemical vapor deposition (CVD), transferring graphene flakes is also a challenge. In addition to these challenges, another serious challenge is cross-sensitivity. Because a sensing environment with a mixture of gases with a similar structure or belonging to the same family may interfere with the response of the sensor, for example, the detection of NO in the exhaled gas and the water molecules carried by the exhaled gas will also affect the sensing. For an ideal sensor, its detection limit should be lower than the range of gases breathed out by a healthy human body and have a clear linear relationship to better detect disease markers in the breath. A correct understanding of the interaction between graphene-based materials and biological systems and their adverse effects is also necessary for the further development and safe use of graphene-based medical exhalation gas diagnosis.

## Figures and Tables

**Table 1:** The relationship between the types and concentrations of exhaled gas and human diseases.

Diseases	Gas	Normal Range	Symptoms Range	References
bromopnea	Hydrogen sulfide	<0.1 ppm	0.1–0.5 ppm	[8]
renal disease	Trimethylamine	<1.75 ppb	1.75–38.02 ppb	[9]
	Isoprene	28–144 ppb	57–329.8 ppb	[9]
diabetes	Acetone	300–900 ppb	≥1800 ppb	[10]
	Ammonia	74–2935 ppb	2935–6770 ppb	[11]
lung cancer	Methanol	157–344 ppb	>344 ppb	[12]
	Ethanol	96–2848 ppb	>2848 ppb	[12]
	Toluene	1–18 ppb	≥18 ppb	[13]
	Benzene	1.1–3.5 ppb	>3.5 ppb	[12]
asthma	Nitric oxide	<25 ppb	>50 ppb	[14]
cancer	Cyclohexane	0.1–15 ppb	>15 ppb	[15]
	Chloroform	<10 ppb	≥10 ppb	[15]

Traditional methods for monitoring breath biomarkers include gas chromatogra mphy–mass spectrometry (GC-MS) [16], selective ion flow tube mass spectrometry (SIFT-MS), proton transfer reaction mass spectrometry (PTR-MS) [17], ion mobility spectroscopy (IMS) [17], enzyme immunoassay [18], polymerase chain reaction, and new antibody microarray technology, etc. [19]. At present, nanomaterials are being developed rapidly to improve the sensitivity, selectivity, and portability of breath detection. For example, Das et al. prepared a sensor of barium hexaferrite oxide nanoparticles (BaFe_12_O_19_) [6] for detecting trace ammonia vapor in human exhalation with a biomarker of kidney disease. Aghaei et al. used first-principles density functional theory calculations and non-equilibrium Green’s function theory to study graphene-like carbon boron nitride (BC_6_N) in human breath analysis as a high-performance volatile organic compound (VOC) sensor [20]. Zhang et al. comprehensively introduced the developments in semiconductor gas sensors made from two-dimensional materials, and their potential application in disease diagnosis [21].

**Table 2:** The detection range and response time of other graphene composite materials to NO, NH3 gas.

Material	Gas	Detection Limit	Response/Restore Time	Reference
Pd–rGO	NO	2–420 ppb	1000 s–1 h	[62]
Sub–graphene–hemin	NO	0.3 nM	47–54 ms	[55]
GQD–Tb^3+^–SOD	NO	600 molecules/mL	500 s	[59]
LPFG coated with GO	NO	0–400 ppm	23.6 min/N.A.	[60]
N–rGO/ZnO	NO	100 ppb	522/303 s	[61]
		300 ppb	478/410 s	
		800 ppb	284/473 s	
Graphene/PS brush	NH_3_	≥4.88 ppb	150 s/N.A.	[63]
PPy	NH_3_	7.6 ppm	105/182 s	[7]
PPy/GO	NH_3_	0.90 ppm	81/116 s	
PPy/rGO	NH_3_	0.035 ppm	72/151 s	
PPy/srGO	NH_3_	0.00020 ppm	48/234 s	
PPy–rGO	NH_3_	10 ppm	~100 s	[64]
PPy/SnO_2_/GNR	NH_3_	≥0.6 ppm	~100 s/200 s	[65]
MoO_x_/GFET	NH_3_	≥310 ppb	356 s	[66]
PUF–PPy–GO	NH_3_	1.1–182 ppm	~7/13 s	[67]
GO–PANI	NH_3_	1 ppm	~5/10 min	[68]

N.A.: not available.

**Table 4:** The detection range and response/recovery time of graphene composite materials to VOC gases.

Materials	Gas	Detection Limit	Response/Recovery Time (s)	References
Au NPs–rGO	TMA	≥5 ppm	~30	[84]
C_60_–g–CNT	ethanol	≥400 ppb	~300/400	[11]
	methanol	≥400 ppb	~300/400	
	acetone	≥400 ppb	~300/400	
	chloroform	≥400 ppb	~300/400	
	toluene	≥400 ppb	~300/400	
	cyclohexane	≥400 ppb	~300/400	
C_60_–g–rGO	ethanol	≥400 ppb	~300/400	[11]
	methanol	≥400 ppb	~300/400	
	acetone	≥400 ppb	~300/400	
	chloroform	≥400 ppb	~300/400	
	toluene	≥400 ppb	~300/400	
	cyclohexane	≥400 ppb	~300/400	
(Parylene C and GO) MS	toluene	100–300 ppm	164/412	[87]
eG	**acetaldehyd**	≥10 ppm	N.A.	[88]
GO thin film	ethanol	>80 ppm	N.A.	
rGO/SnO_2_	acetone	0.25–30 ppm	24/30 (5 ppm)	[89]
Sn_x_Ti_1−x_O_2_/GO	toluene	100 ppb	N.A.	[90]
	acetone	200 ppb	N.A.	
Ag/Fe_3_O_4_/rGO	acetone	35.81–50 ppm	~50/70	[91]
SiNW/rGO	**acetaldehyd**	1 ppm	30/180	[92]
	cyclohexane	1 ppm	30/60~120	

N.A.: not available.
